# ALDH1A3 Accelerates Pancreatic Cancer Metastasis by Promoting Glucose Metabolism

**DOI:** 10.3389/fonc.2020.00915

**Published:** 2020-06-16

**Authors:** Shuang Nie, Xuetian Qian, Mengyue Shi, Hongzhen Li, Chunyan Peng, Xiwei Ding, Shu Zhang, Bin Zhang, Guifang Xu, Ying Lv, Lei Wang, Helmut Friess, Bo Kong, Xiaoping Zou, Shanshan Shen

**Affiliations:** ^1^Department of Gastroenterology, The Affiliated Drum Tower Hospital of Nanjing University Medical School, Nanjing, China; ^2^Department of Gastroenterology, Nanjing Drum Tower Hospital, Clinical College of Nanjing Medical University, Nanjing, China; ^3^Department of Surgery, Klinikum rechts der Isar, School of Medicine, Technical University of Munich (TUM), Munich, Germany

**Keywords:** pancreatic ductal adenocarcinoma, ALDH1A3, PPARγ, HK2, tumor metastasis, glycolysis

## Abstract

**Background:** The aldehyde dehydrogenase 1 family member A3 (ALDH1A3) is a key enzyme associated with a variety of metabolic processes, including glucose metabolism. We recently uncovered that glucose metabolism played an essential role in promoting metastasis of pancreatic ductal adenocarcinoma (PDAC). As ALDH1A3 labels an aggressive subtype of PDAC, we hypothesized that ALDH1A3 functionally promoted PDAC metastasis via its metabolic effect on glucose metabolism.

**Methods:** Expression of ALDH1A3 was detected in human PDAC tissues by immunohistochemistry. ALDH1A3 was knocked down or overexpressed in PDAC cells by either shRNA or overexpression vector. The functional roles of ALDH1A3 were characterized *in vitro* and *in vivo*. Transcriptional profiling via RNA-sequencing was used to explore the possible underlying molecular mechanisms. Glucose uptake, extracellular lactate, and ATP production were measured to access the metabolic influence of ALDH1A3 on PDAC cells.

**Results:** ALDH1A3 was associated with poor prognosis in PDAC patients. Functionally, ALDH1A3 promoted PDAC metastasis *in vitro* and *in vivo*. Further studies revealed that ALDH1A3 activated PI3K/AKT/mTOR signaling pathway and its downstream target-PPARγ (peroxisome proliferator-activated receptor gamma). This led to increase the expression of HK2 (hexokinase 2), which subsequently enhanced the glycolysis in PDAC cells. Additionally, the pharmacological inhibition of PPARγ activity in ALDH1A3-positive cells impaired glycolytic genes expression, PI3K/AKT/mTOR activity and cellular glycolysis.

**Conclusions:** ALDH1A3 promotes PDAC metastasis via its metabolic influence on glucose metabolism. PPARγ and its downstream PI3K/AKT/mTOR signaling pathway maybe involved in this process.

## Introduction

Pancreatic ductal adenocarcinoma (PDAC) is an extremely aggressive disease with dismal prognosis (1). Despite enormous studies in the understanding of molecular tumor biology, the overall survival rate of PDAC patients has remained unchanged for the past 20 years (2, 3). This may be partially attributed to the fact that PDAC is a complex, heterogenic disease with extensive variations in genetic, clinical, and histological profiles. A variety of molecular subtypes with distinctive tumor biology seem to exist (4), thus it is an unmet need to sub-classify PDAC into clinically relevant subtypes and subsequently to develop targeted therapies accordingly. In this regard, we previously used a reverse-translational approach to define PDAC subtypes in mice (5–9) and generated several novel transgenic mouse models of PDAC entities with different potency of aggressiveness. Detailed analyses revealed that metastasis was driven by the Kras^G12D^/Mek/mTOR signaling axis, for which ALDH1A3 (aldehyde dehydrogenase family 1, subfamily A3) constituted a specific marker (8). Furthermore, ALDH1A3 labeled an aggressive subtype of human PDAC with very short overall survival.

Metabolism reprogramming is one of characteristic hallmarks in cancer cells. In contrast with normal cells, cancer cells primarily rely on aerobic glycolysis for glucose metabolism even under normoxia, which is known as the Warburg effect (10). Aerobic glycolysis could efficiently generate the energy and macromolecules required for cell growth, thereby fueling the rapid growth and proliferation observed in tumors (11). Aberrant regulation of glycolysis-related genes is partly responsible for metabolic shift to aerobic glycolysis to facilitate cancer progression (12, 13). Notably, ALDH1A3 is a key enzyme involved in a variety of metabolic processes, including glucose metabolism (14, 15). Thus, we hypothesized that ALDH1A3 might promote PDAC progression via regulating glucose metabolism. To confirm this, the expression of ALDH1A3 in human PDAC tissues was detected primarily. Furthermore, we generated human PDAC cell lines with different expression levels of ALDH1A3. Then *in vitro* and *in vivo* functions of ALDH1A3 were tested by PDAC metastasis and glucose metabolism. Transcriptional profiling via RNA-sequencing was applied to explore the possible underlying molecular mechanisms.

## Results

### High Expression of ALDH1A3 Is Correlated With Poor Prognosis in PDAC

**Figure 1:**
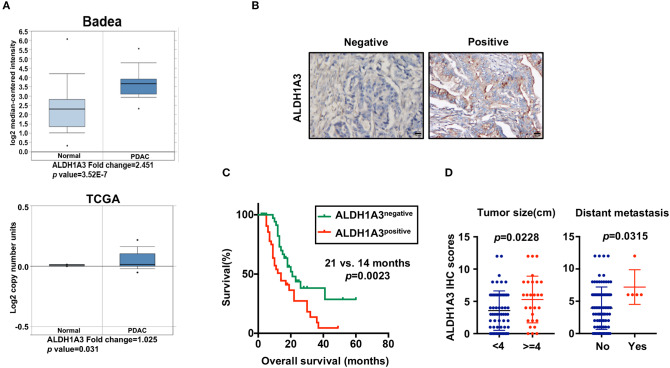
High expression of ALDH1A3 is correlated with poor prognosis in PDAC. **(A)** The expression of ALDH1A3 increased in pancreatic cancer tissue compared to adjacent normal pancreatic tissue by Oncomine dataset analysis. **(B)** ALDH1A3 immunostaining signals were primarily detected mainly in cytoplasm, as well as in stroma partly. Bar:20 μm. **(C)** High expression of the ALDH1A3 in cancer tissues was associated with shorter overall survival time in the PDAC patients (*p* = 0.0023). **(D)** The ALDH1A3 expression levels were significantly associated with tumor size (*p* = 0.0228) and distant metastasis (*p* = 0.0315).

To uncover the relationship between ALDH1A3 expression and clinical parameters in human PDAC, we first analyzed four previously published datasets about the expression of ALDH1A3 in PDAC vs. normal tissues from Oncomine. The results revealed that in two datasets ALDH1A3 was upregulated in PDAC tissues compared to the corresponding adjacent non-tumor tissues (Figure 1A). Though the other two datasets showed no significant change, the increased expression of ALDH1A3 mRNA levels in bulk PDAC tissues was also experimentally validated using QRT-PCR assay (8). So far, the reason for this disparity is unclear. We speculate that this might be due to the lack of sufficient case numbers of these databases. Furthermore, we investigated the expression ALDH1A3 in a cohort of 88 human PDAC tissues by immunohistochemical staining. This analysis revealed that ALDH1A3 mainly express in ductal epithelial cells, as well as in stromal cells partly. And the staining of which was primarily detected in cytoplasm (Figure 1B). Kaplan–Meier analysis revealed that patients with high expression of ALDH1A3 were associated with shorter overall survival time (*p* = 0.0023, Figure 1C). The median overall survival time was 21 months in the ALDH1A3-negative expression group and 14 months in the ALDH1A3-positive expression group. The HR of ALDH1A3-negative was 0.4713 (95% CI, 0.2473–0.8044). Moreover, ALDH1A3 expression levels were significantly associated with tumor size (*p* = 0.0228) and distant metastasis (*p* = 0.0315, Figure 1D). Additionally, chi-square test indicated the higher proportion of ALDH1A3-positive expression cases in patients with tumor size larger than 4 cm (*p* = 0.046) and in patients with distant metastasis (*p* = 0.028) in PDAC patients (Table 1). However, the number of cases with metastasis was low, further studies in this aspect is required.

**Table 1:** The relationship between ALDH1A3 expression and clinicopathological features of PDAC patients.

**Clinicopathological features**	**Numbers**	**ALDH1A3**		**χ^2^**	***P*-value**
		**Negative**	**Positive**		
**Sex**					
Male	51	21	30	2.086	0.149
Female	37	21	16		
**Age**					
<60	57	27	30	0.008	0.927
≥60	31	15	16		
**TNM stage**					
I/II	79	39	40	0.833	0.362
III/IV	9	3	6		
**T-classification**					
T1+T2	37	20	17	1.024	0.311
T3+T4	51	22	29		
**Tumor size (cm)**					
<4	60	33	27	3.998	0.046
≥4	28	9	19		
**Differentiation degree**					
Low	32	15	17	0.015	0.904
Moderate/High	56	27	29		
**Lymph-node metastasis**					
Absent	51	24	27	0.022	0.883
Present	37	18	19		
**Vascular invasion**					
Absent	64	30	34	0.068	0.794
Present	24	12	12		
**Diabetes**					
Absent	61	26	35	2.076	0.150
Present	27	16	11		
**Distant metastasis**					
Absent	83	42	41	4.840	0.028
Present	5	0	5		

### ALDH1A3 Promotes Migration and Metastasis in PDAC

**Figure 2:**
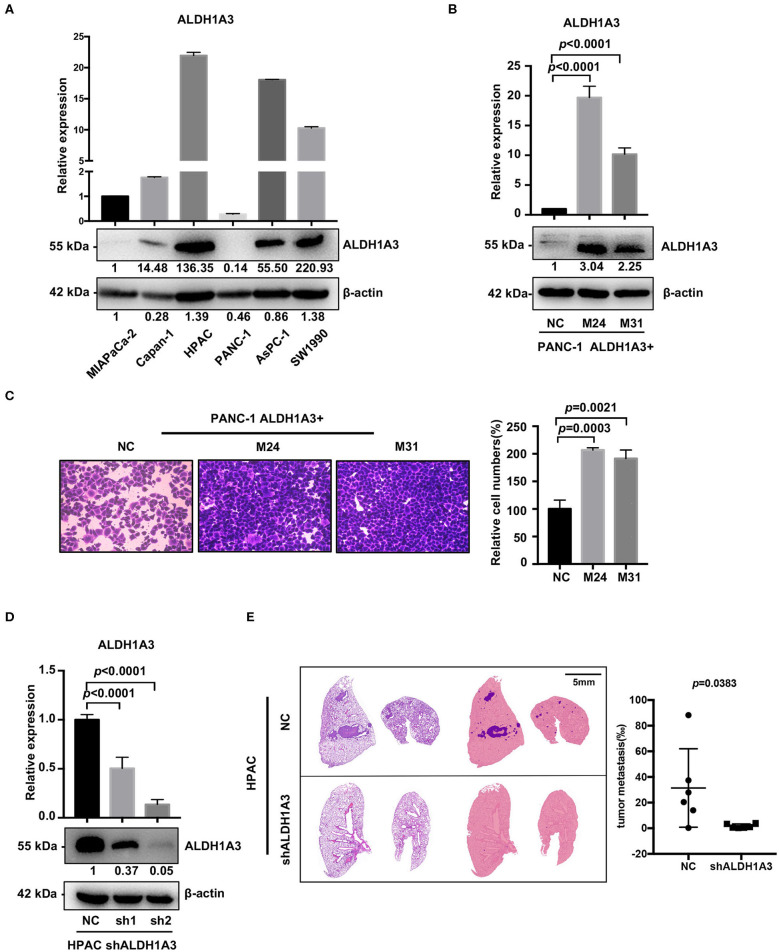
ALDH1A3 promotes migration *in vitro* and metastasis *in vivo*. **(A)** ALDH1A3 expression was detected in six human pancreatic cancer cell lines at mRNA and protein levels. **(B)** The overexpression of ALDH1A3 in monoclonal cells (named as M24 and M31) at mRNA and protein levels. **(C)** The overexpression of ALDH1A3 in PANC-1 cells increased the number of migrated cells in transwell assay at 24 h after cell seeding. **(D)** Transfection efficiency of the lentiviral vector expressing shRNAs targeting ALDH1A3 in HPAC cells at mRNA and protein levels. **(E)** Knock-down of ALDH1A3 in HPAC cells reduced the area proportion of metastatic lesions in the largest section of each lung sample 5 weeks after cell injection.

To investigate the potential effects of ALDH1A3 on migration and metastasis of PDAC, we further studied the role of ALDH1A3 in human PDAC cell lines. First, we detected mRNA and protein levels of ALDH1A3 in six human PDAC cell lines, among which, PANC-1, MIAPaCa-2, Capan-1 had relatively low expression of ALDH1A3 (defined as ALDH1A3-negative cells) and AsPC-1, HPAC, SW1990 had relatively high expression of ALDH1A3 (defined as ALDH1A3-positive cells) (Figure 2A). Thus, we chose PANC-1 and HPAC cells for further overexpression and knockdown assays, respectively.

To generate overexpression of ALDH1A3 in ALDH1A3-negative cells, TrueORF cDNA transfection was conducted in PANC-1 cells, followed by monoclonal collection (named as M24 and M31) for stable overexpression (Figure 2B). Due to PANC-1 cells did not readily form lung metastasis tumors in our studies, transwell assay *in vitro* was conducted to test the potential impact of ALDH1A3 on migration and the results showed that the overexpression of ALDH1A3 significantly promoted the migration ability of PANC-1 cells (Figure 2C).

To achieve stable knockdown of ALDH1A3 in HPAC cells, two shRNAs targeting ALDH1A3 was constructed into a lentiviral vector. HPAC cells were transfected with the two shRNAs and knockdown efficiency was determined by qRT-PCR and western-blotting analysis (Figure 2D). Both shRNAs achieved satisfactory knockdown efficiency and was used for further study. To evaluate the effect of ALDH1A3 on PDAC metastasis, a lung-metastasis xenograft mouse model of HPAC cells was conducted. The area of metastatic lesions in lung was calculated and analyzed 5 weeks after intravenous inoculation. As shown in Figure 2E, knockdown of ALDH1A3 significantly reduced the area proportion of metastatic lesions (*p* = 0.0383).

### ALDH1A3 Promotes the Expression of Key Enzymes in Glycolysis

**Figure 3:**
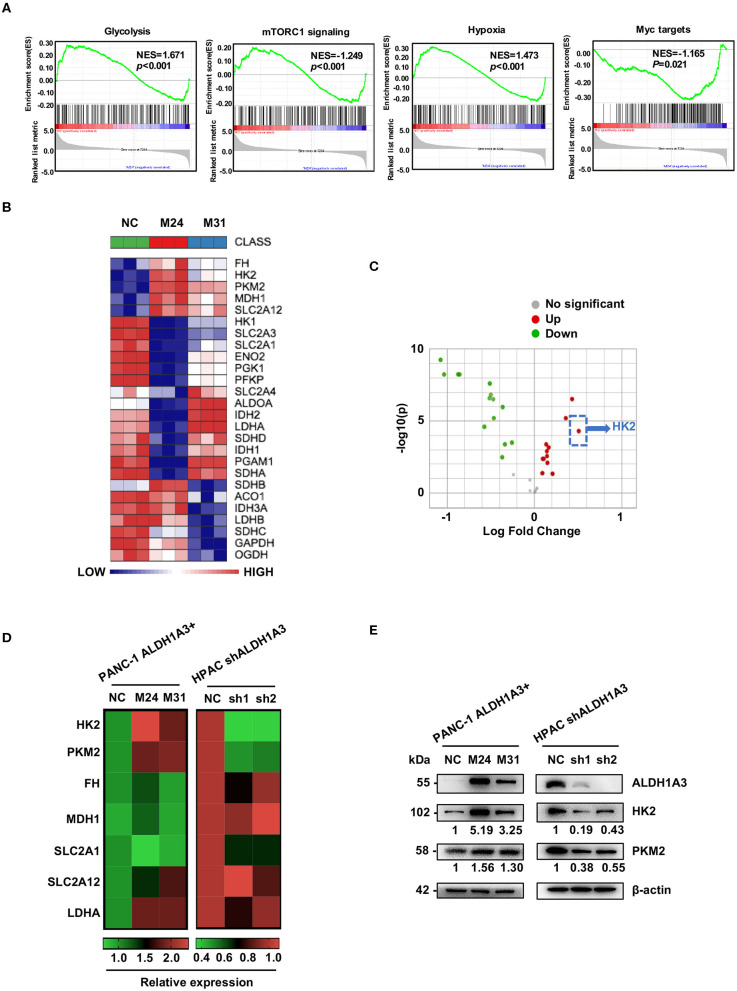
ALDH1A3 promotes the expression of key enzymes of glycolysis. **(A)** GSEA using hallmark gene sets was performed to compare the ALDH1A3 overexpression group and negative control group. NES, normalized enrichment score. **(B)** A heat map showing the expression of glycolysis-related genes across ALDH1A3 overexpression samples and negative control samples. **(C)** A volcano plot showed the fold change of glycolysis-related genes after the overexpression of ALDH1A3 in PANC-1 cells. **(D)** QRT-PCR results showed the relative mRNA levels of glycolysis-related genes in PANC-1 cells conducted by ALDH1A3 overexpression or in HPAC cells conducted by ALDH1A3 knock-down. **(E)** Western-blotting results showed the protein level of HK2 and PKM2 expression in PANC-1 cells conducted by ALDH1A3 overexpression or in HPAC cells conducted by ALDH1A3 knock-down.

To gain an insight into the mechanisms by which ALDH1A3 promoted PDAC metastasis, the gene expression in the ALDH1A3-overexpression PDAC cells (M24 and M31) and negative control cells was profiled by RNA sequencing and analyzed by gene set enrichment analysis (GSEA). RNA sequencing results revealed that 2,131 genes were differentially expressed according to diverse ALDH1A3 expression levels. Additionally, GSEA results showed that these genes were accumulated to metabolic processes, including glycolysis, mTORC1 signaling, hypoxia and Myc targets, suggesting that ALDH1A3 may alter the glycolytic flux in PDAC cells (Figure 3A). Furthermore, several key genes in glucose metabolism including HK2 (Hexokinase2), PKM2 (Pyruvate kinase M2), FH (Fumarate hydratase), MDH1 (Malate dehydrogenase 1), SLC2A12 (Solute carrier family 2 member 12), SLC2A1 (Solute carrier family 2 member 1), and LDHA (L-lactate dehydrogenase A chain) were significantly altered in the ALDH1A3-overexpression PDAC cells compared to negative control cells (Figure 3B). Moreover, the key enzyme of glycolysis-HK2 showed the largest fold change among those up-regulated genes (Figure 3C). Alteration of HK2, PKM2, FH, MDH1, and SLC2A12 were further confirmed by qRT-PCR (Figure 3D) both in PANC-1 and HPAC cells. However, the results indicated that only the expression of HK2 and PKM2 decreased when ALDH1A3 was down-regulated in HPAC cells (Figure 3D). This was also confirmed at the protein expression level by western-blotting (Figure 3E). HK2, PKM2, and LDHA are key enzyme in glycolysis, and other altered genes mainly locate in mitochondria, catalyzing the substrates to facilitate a transition step in the production of energy. Though these genes altered significantly after ALDH1A3-overexpression in PANC-1 cells, most of them (except for HK2 and PKM2) did not altered after ALDH1A3-knockdown in HPAC cells. Thus, we focused on the regulation of HK2 and PKM2 in the study.

### ALDH1A3 Enhances PDAC Glycolysis and Activates the PI3K/AKT/mTOR Pathway

**Figure 4:**
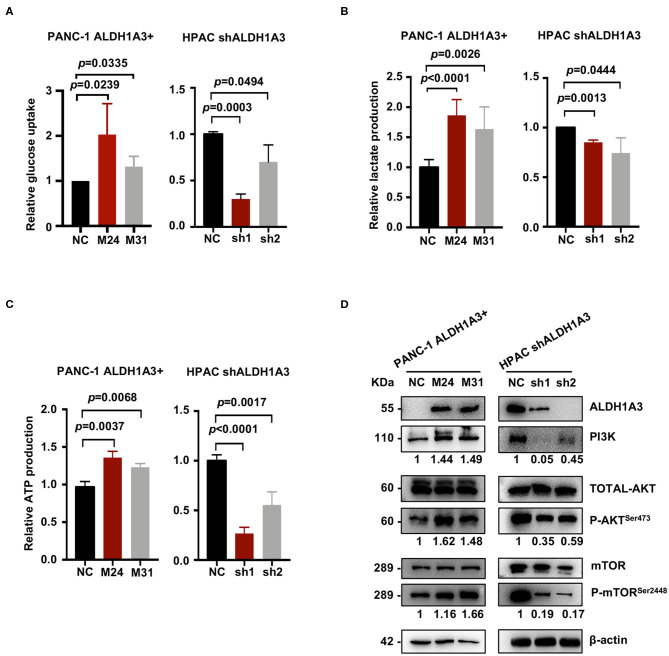
ALDH1A3 enhances PDAC glycolysis by activating the PI3K/AKT/mTOR pathway. **(A–C)** Compared to the control group cells, the PANC-1 cells treated with ALDH1A3-overexpression showed a significant increment in glucose uptake, lactate production, and ATP production, while the HPAC cells treated with ALDH1A3-knockdown showed a decrease in glucose uptake, lactate production and ATP production. **(D)** ALDH1A3 led to significant enhancement of PI3K/AKT signaling including its downstream targets mTOR.

To further investigate the effects of ALDH1A3 on PDAC cells, a number of metabolic assays including glycose uptake, lactate production, and ATP production assays were performed to measure glycolytic activity. Compared to negative control cells, the PANC-1 cells with ALDH1A3-overexpression showed a significant elevation in glucose uptake, lactate production, and ATP production, while the HPAC cells with ALDH1A3-knockdown showed a decrease in glucose uptake, lactate production, and ATP production (Figures 4A–C). Moreover, overexpression of ALDH1A3 in PANC-1 cells decreased the production of ROS both in cells and in mitochondria (Supplementary Figures 1A,B). The results of these functional assays implied that ALDH1A3 induced glycolysis activity in human PDAC cells.

To further understand the molecular mechanisms underlying the regulation of ALDH1A3 on glycolysis, Kyoto Encyclopedia of Genes and Genomes (KEGG) pathway enrichment was performed to detect the potentially involved signaling pathways. Several glucose metabolism-related pathways were involved, such as AGE-RAGE, Glucagon signaling pathway, and Glycolysis/Gluconeogenesis, etc. Notably, these pathways converged on the PI3K/AKT pathway. Indeed, western-blotting results confirmed that the ALDH1A3 overexpression in PANC-1 cells led to a significant enhanced activity in PI3K/AKT signaling and its downstream target-mTOR signaling, while the ALDH1A3 knock-down in HPAC cells led to a decreased activity in PI3K/AKT/mTOR signaling pathway (Figure 4D).

### ALDH1A3 Promotes Glycolysis by Regulating the Expression of HK2

**Figure 5:**
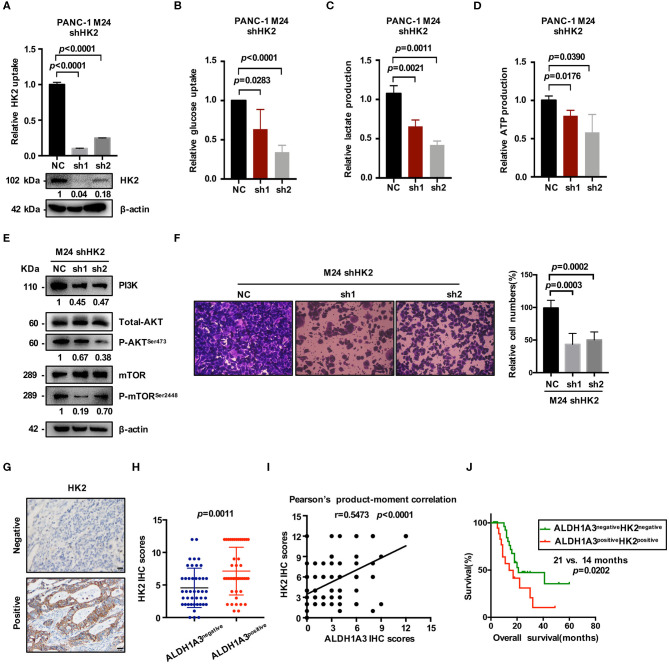
ALDH1A3 promotes glycolysis by regulating the expression of HK2. **(A)** The knockdown efficacy of HK2 in ALDH1A3-overexpression cells (M24 and M31) was guaranteed at both mRNA and protein level. **(B–D)** After HK2 was knocked down in M24 cells, glucose uptake, lactate production and ATP production decreased in return. **(E)** Knockdown of HK2 inhibited PI3K/AKT/mTOR signaling in M24 cells. **(F)** The migration ability of ALDH1A3 could be impaired by HK2 knockdown in M24 cells. **(G)** In PDAC tissues, HK2 immunostaining signals were primarily detected in the cytoplasm. Bar: 20 μm. **(H,I)** The expression of HK2 was higher in ALDH1A3-positive PDAC tissues and correlated positively with the expression of ALDH1A3 in human PDAC tumor tissues. **(J)** The PDAC patients with both ALDH1A3 and HK2 positive expression suffered from poorer overall survival than that with both ALDH1A3 and HK2 negative expression (*p* = 0.0202).

HK2, as the key enzyme of glycolysis, was significantly up-regulated by ALDH1A3-overexpression. To confirm the connection between ALDH1A3 and HK2, we further down-regulated HK2 by lentiviral-mediated shRNA expression in ALDH1A3-overexpression monoclonal PANC-1 cells (M24). The knockdown efficacy was confirmed by both mRNA and protein level (Figure 5A). The results showed that glucose uptake, lactate production, and ATP production decreased upon HK2 knockdown in M24 cells. In addition, it also reversed the increased glycolysis induced by ALDH1A3-overexpression (Figures 5B–D). Furthermore, the activity of PI3K/AKT/mTOR signaling pathway was also inhibited by HK2 knockdown in M24 cells (Figure 5E). Additionally, the transwell assay result showed that the migration ability of ALDH1A3 could be impaired by HK2 knockdown in M24 cells (Figure 5F).

Moreover, the correlation between HK2 and ALDH1A3 was also confirmed by immunohistochemical staining in PDAC tissues. HK2 immunostaining signals were primarily detected in the cytoplasm (Figure 5G). The result showed that the expression of HK2 was higher in ALDH1A3-positive PDAC tissues, and correlated positively with the expression of ALDH1A3 in human PDAC tumor tissues (Figures 5H,I). Moreover, the PDAC patients with both ALDH1A3 and HK2-positive staining had a shorter overall survival than patients with both ALDH1A3 and HK2-negative staining (*p* = 0.0202, Figure 5J).

### ALDH1A3 Activates the PI3K/AKT Signaling Pathway and Up-Regulates HK2 by Crosstalking With PPARγ Pathway

**Figure 6:**
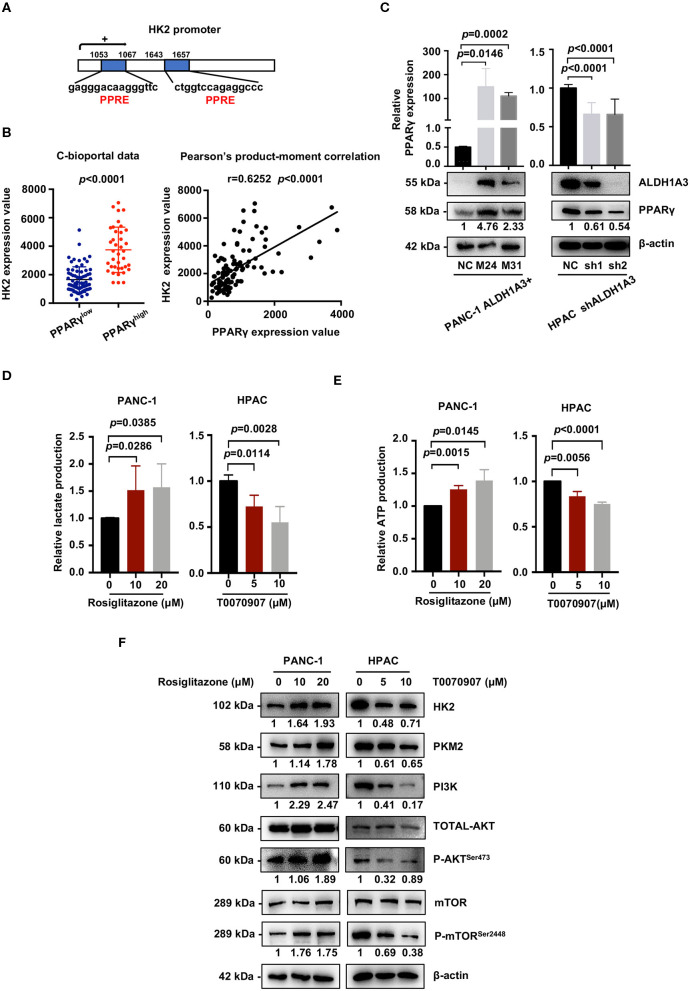
ALDH1A3 activates the PI3K/AKT pathway and up-regulates HK2 by crosstalking with PPARγ. **(A)** The promoter regions of HK2 genes contained several PPAR response elements (PPRE). **(B)** Analyses of data on the cBioPortal showed the expression of HK2 was higher in PPARγ-high expression group compared to that in PPARγ-low expression group (*p* < 0.0001), and the expression of PPARγ and HK2 showed significant positive correlation (*p* < 0.0001). **(C)** ALDH1A3 overexpression in PANC-1 cells up-regulated PPARγ expression while ALDH1A3 knockdown in HPAC cells decreased the expression of PPARγ. **(D,E)** The PPARγ agonist-Rosiglitazone used in PANC-1 cells could increase lactate production and ATP production, while the PPARγ inhibitor-T0070907 used in HPAC cells could decrease lactate production and ATP production. **(F)** The PPARγ agonist-Rosiglitazone used in PANC-1 cells could up-regulate the expression of HK2 and PKM2 and activate PI3K/AKT/mTOR signaling pathway, while the PPARγ inhibitor-T0070907 used in HPAC cells could down-regulate the expression of HK2 and PKM2 and suppress PI3K/AKT/mTOR signaling pathway.

We next investigated the mechanism of how ALDH1A3 activated PI3K/AKT/mTOR pathway and promoted HK2-mediated glycolysis. Here, a previous study reported PPARγ played an important role in the transcription of glycolytic isozyme genes-HK2 (16). ALDH1A3 is an important aldehyde metabolic enzyme responsible for retinoic acid signaling. To confirm it, by *in silico* analysis, we found that the promoter regions of HK2 genes contained several PPAR response elements (PPRE, Figure 6A). Consistently, by analyzing the data on the cBioPortal, the expression HK2 was higher in PPARγ-high expression group compared to that in PPARγ-low expression group (*p* < 0.0001, Figure 6B). Moreover, the expression of PPARγ and HK2 showed a significantly positive correlation (*p* < 0.0001, Figure 6B). In line, ALDH1A3 overexpression in PANC-1 cells increased the PPARγ expression while ALDH1A3 down-regulation in HPAC cells decreased the PPARγ expression (Figure 6C).

To further investigate the role of PPARγ in ALDH1A3-induced glycolysis in PDAC cells, a PPARγ agonist-rosiglitazone and a PPARγ inhibitor-T0070907 were used in PANC-1 (ALDH1A3-negative) and HPAC cells (ALDH1A3-positive), respectively. The results revealed that the rosiglitazone treatment (PPARγ activation) increased the lactate and ATP production in PANC-1 cells (Figures 6D,E). Meanwhile, the expression of HK2 and PKM2 increased, and the PI3K/AKT/mTOR signaling pathway was activated after the rosiglitazone treatment (Figure 6F). However, the T0070907 treatment (PPARγ inhibition) decreased the lactate and ATP production in HPAC cells (Figures 6D,E). Correspondingly, the expression of HK2 and PKM2 was down-regulated and PI3K/AKT/mTOR signaling was inhibited (Figure 6F). However, the glucose uptake was not affected by neither PPARγ agonist nor inhibitor, which might due to the concept that PPARγ could influence other steps of glycolysis, not only depend on uptake of glucose to regulate tumor metabolism. Thus, we concluded that ALDH1A3 promoted glycolysis and regulated the expression of HK2 via transcription factor PPARγ.

## Discussion

Previously, we generated a number of PDAC entities with different potency of aggressiveness by using a set of transgenic mouse models (5–9). Notably, in the context of Tsc1 haploinsufficiency, Kras^G12D^ preferably activated mTOR signaling via the Mek/Erk cascade, contributing to a PDAC subtype with high-metastasis potential (8). Expression analysis identified ALDH1A3 as a key target gene of Kras^G12D^/Mek/mTOR, both on the expression and the function levels (8). Thus, we hypothesized that ALDH1A3 played an important role in the progression of this aggressive PDAC subtype. However, the mechanisms of ALDH1A3 in PDAC progression remain unknown (17). Previous studies suggest that ALDH1A3 is closely associated with the colony formation, sphere formation, tumorigenesis, and angiogenic activity of cancer cells (15). High expression of ALDH1A3 predicts poor prognosis in glioma (18), melanoma (19), glioblastoma (20), and breast cancer (21). However, the role of ALDH1A3 has not so far been reported under physiological circumstances in PDAC cells. Our clinical study results showed that high expression of ALDH1A3 was correlated positively with tumor size and distant metastasis, and predicted poor prognosis in PDAC. *In vitro* and *in vivo* studies further confirmed that ALDH1A3 could promote migration and metastasis, respectively. These data suggest a promotional role of ALDH1A3 on PDAC progression.

Previous studies have reported that ALDH1A3 was a key enzyme associated with a variety of metabolic processes, including glucose metabolism (15). Our transcriptomic and metabolic analyses revealed that the metastasis-promoting role of ALDH1A3 was largely dependent on glycolysis and the key glycolytic enzyme HK2. Among glycolytic enzymes, HK2 particularly favors aerobic glycolysis because of its kinetic features and intracellular distribution (16). Accumulating studies have confirmed that HK2 expression was significantly up-regulated in a variety of malignant tumors, including PDAC, and it could directly promote metastasis via regulation of glycolysis (22, 23). Additionally, during ALDH1A3-induced metabolic reprograming, we found that ALDH1A3 activated PI3K/AKT/mTOR signaling by crosstalking with transcription factor PPARγ. Enhanced PI3K/AKT signaling promotes the metabolic reprogramming by either promoting the activity of hexokinase in driving glycolysis or activating the AKT-dependent mTOR signaling to enhance the expression of glycolysis-related genes (24, 25). PPARγ is a member of the nuclear receptor superfamily that regulates metabolism, inflammation, and cellular growth and differentiation. It has been implicated in the carcinogenesis and progression of various solid tumors, including PDAC. Inhibition of PPARγ could suppress hepatic metastasis of PDAC in mice (26). Notably, a recent study found that PPAR response elements (PPRE) existed in the promoter of ALDH1A3, and ALDH1A3 could up-regulate PPARγ in lung cancer cells (27), consistent with our observation in PDAC cells. Furthermore, it has been previously demonstrated that PPARγ could activate PI3K/AKT pathway, which then regulated the novel and direct targets HK2 and PKM2, leading to hepatic steatosis and cell proliferation (16). In our study, suppressing PPARγ in ALDH1A3-positive PDAC cells could inhibit glycolysis, the expression of HK2 and PI3K/AKT/mTOR signaling pathway correspondingly. Thus, it is likely that ALDH1A3 induces glucose metabolism in PDAC by crosstalking with PPARγ.

In conclusion, our results demonstrate that ALDH1A3 promotes PDAC metastasis via its metabolic influence on glucose metabolism. PPARγ and its downstream PI3K/AKT/mTOR signaling pathway seem to be involved in this process. Taken together, our results provide new insight that targeting ALDH1A3 might constitute a new approach for PDAC treatment.

## Methods and Materials

### Data Mining Using Oncomine and TCGA

ALDH1A3 gene expression in PDAC tissues and adjacent normal tissues was analyzed using microarray gene expression datasets deposited in the Oncomine database (https://www.oncomine.org). A combined filter was applied to display the corresponding datasets. The Cancer Type was defined as Pancreatic Cancer and Data Type was mRNA, and Analysis Type was Cancer vs. Normal Analysis.

The expression of PPARγ and HK2 data for TCGA pancreatic adenocarcinoma (provisional) patients (*n* = 141) were downloaded and filtrated from the cBioPortal website (http://www.cbioportal.org). The cutoff value for PPARγ mRNA expression (RNA Seq V2 RSEM) was the average 922, and the cutoff value for HK2 mRNA expression was the average 2,603.

### Human PDAC Tissue Array Analysis

The experiments utilizing human samples were approved by the Ethical Committee of Medical Research, the Affiliated Drum Tower Hospital of Nanjing University Medical School (202001301). The patient cohort of human pancreatic tissue array containing 88 PDAC specimens was also obtained from Drum Tower Hospital from August 2010 to July 2018. Patients had not received radiotherapy, chemotherapy, or other related anti-tumor therapies before surgery.

### Immunohistochemistry

For histological analyses, PDAC tissues from patients were fixed in 10% buffered formalin (Sigma) and embedded in paraffin. Paraffin sections were then processed for either hematoxylin and eosin (H&E) staining or immunohistochemistry (28). Specific antibodies used for immunohistochemistry were: ALDH1A3 (1:100, Arigo, #14766), HK2 (1:200, Cell Signaling Technology, #2867s). The proportion of positive stained areas was evaluated as follows: 0 for <5%, 1 for 5–25%, 2 for 25–50%, 3 for 50–75%, and 4 for ≥75%. The intensity of staining was scored as 0, 1, 2, and 3 for the representation of no color, yellow, brown, and dark brown. The final scores were obtained by multiplying the extent of positivity and intensity scores, producing a range from 0 to 12. For ALDH1A3, no <4 points are defined as positive. For HK2, meeting 6 points is positive. The stained slides were evaluated by two experienced pathologists independently.

### Cell Culture and Reagents

Human pancreatic cancer cell lines PANC-1, HPAC, AsPC-1, SW1990, MIAPaCa-2, Capan-1 were gifted from department of surgery, Klinikum rechts der Isar, Technical University of Munich. All cell lines used in this study are considered to be identical to the reference cell line in the Cell Bank STR database. Mycoplasma testing is also negative.

All cell lines were cultured in a suggested medium according to ATCC protocols in a humidified incubator at 37°C with 5% CO2. HPAC cells with high ALDH1A3 expression and PANC-1 cells with low ALDH1A3 expression were selected for further study.

Rosiglitazone (APExBIO, #A4303), a therapeutic drug for diabetes, was used as an agonist of PPARγ. T0070907(Selleck Chemicals, #S2871) was used as an antagonist of PPARγ. Cells were treated with rosiglitazone (10 and 20 μM) and T0070907 (5 and 10 μM) for 72 h, and then managed for next research steps.

### Plasmid Transfection

For ALDH1A3 overexpression transfection, PANC-1 cells with low ALDH1A3 expression was transfected with 1 μg of either human cDNA ORF clone (OriGene, #RC209656) or control ORF clone (OriGene, #PS100001). 1.5 mg/mL geneticin (Sigma-Aldrich, #A1720) was supplemented in culture medium for selection.

### Lentivirus Transfection

ALDH1A3-shRNA lentiviral vector was constructed by GeneChem Co., Ltd., (Shanghai, China) from GV248 vector (hU6-MCS-Ubiquitin-EGFP-IRES-puromycin). The targeting sequence of ALDH1A3-shRNA was GCAACCAATACTGAAGTTCAA (sh1), GAGCAGGTCTACTCTGAGTTT (sh2). Empty vector was used as the negative control. To generate stable ALDH1A3-knockdown cells, HPAC cells were placed in a 6-well plate at a density of 1 × 10^5^ cells/well the day before infection. The next day 5 × 10^5^ units of lentivirus were added in cell culture medium supplied with 5 μg/ml polybrene to initiate infection. Viruses were removed 12 h after infection and fresh cell culture medium was added. Three days after transfection, 1 μg/ml puromycin was added into the cell culture medium to generate stable ALDH1A3-knockdown HPAC cell line.

HK2-shRNA lentiviral vector was constructed by GeneChem Co., Ltd., (Shanghai, China) from GV248 vector (hU6-MCS-Ubiquitin-EGFP-IRES-puromycin). The targeting sequence of HK2-shRNA was CTGGCTAACTTCATGGATA (sh1), GTAACATTCTCATCGATTT (sh2). Transfection method was the same as above.

### RNA Extraction and Quantitative Real-Time PCR

Total RNA was isolated using Trizol reagent (Takara) according to the manufacturer's instructions. Reverse transcription reactions were performed on 1 μg of total RNA with the PrimeScript™ RT Master Mix (Takara). Quantitative PCR was performed in a total reaction volume of 20 μL using SYBR® Advantage® qPCR Premix (Takara) according to the manufacturer's recommendations. Reactions were run in triplicate in three independent experiments. The 2^−ΔΔ*CT*^ method was used to determine the relative levels of mRNA expression between experimental samples and controls. The experiments were performed three biological repeats and three technical replicates. Primers were listed as following. ALDH1A3 forward: 5′-ACTCTGAGTTTGTCAGGCGG-3′, ALDH1A3 reverse: 5′-CACTGGCCCGAAAATCTCCT-3′; HK2 forward: 5′-AGGAGAACAAAGGCGAGGAG-3′, HK2 reverse: 5′-GCCGCACGGTCTTATGTAGA-3′; PKM2 forward: 5′-GGTGGAAAATGGTGGCTCCT-3′, PKM2 reverse: 5′-TGCGGATGAATGACGCAAAC-3′; FH forward: 5′-GCACCATGTACCGAGCACTT-3′, FH reverse: 5′-CCGGAAGGAATTTTGGCTTGC-3′; MDH1 forward: 5′-TGCTGTCATCAAGGCTCGAA-3′, MDH1 reverse: 5′-ACAAACTCTCCCTCTGGGGT-3′; GLUT1 forward: 5′-TGGCATCAACGCTGTCTTCT-3′, GLUT1 reverse: 5′-AACAGCGACACGACAGTGAA-3′; SLC2A12 forward: 5′-TTTCTTCCTCCAAGCCCTCG-3′, SLC2A12 reverse: 5′-GGGTCCGCATGTTGTCTTTT-3′; LDHA forward: 5′-ATGGCAACTCTAAAGGATCAGC-3′; LDHA reverse: 5′-CCAACCCCAACAACTGTAATCT-3′; PPARγ forward: 5′-GGTTTCTTCCGGAGAACAATCA-3′; PPARγ reverse: 5′-ATCCCCACTGCAAGGCATTT-3′; β-actin forward: 5′-CTACGTCGCCCTGGACTTCGAGC-3′; β-actin reverse: 5′-GATGGAGCCGCCGATCCACACGG-3′.

### Western Blotting

Cell and tissues lysates were collected as previously described (29). Protein concentrations were determined using BCA Assay (Beyotime Biotechnology). Equal amounts of protein were separated with 8–12% SDS-PAGE and then electrophoretically transferred onto a polyvinylidene difluoride membrane (Millipore, Billerica, MA, USA). TBST containing with 5% non-fat milk or bovine serum albumin was used to block non-specific binding for 2 h at room temperature. Then, membranes were incubated with primary antibodies according to the instructions overnight at 4°C followed by appropriate secondary antibodies. Signals generated by enhanced chemiluminescence (Millipore) were recorded with a CCD camera (Tanon, Shanghai). The experiments were performed three biological repeats. Primary and secondary antibodies included: ALDH1A3 (1:1,000, Sigma-Aldrich, #HPA046271), HK2 (1:1,000, Cell Signaling Technology, #2867), PKM2 (1:1,000, Cell Signaling Technology, #4053), PPARγ (1:1,000, Santa Cruze, #sc-7273), mTOR (1:1,000, Cell Signaling Technology, #2983), p-mTOR (1:1,000, Cell Signaling Technology, #5536), PI3 Kinase p110α (1:1,000, Cell Signaling Technology, #4249), Akt (1:1,000, Cell Signaling Technology, #2920), p-Akt (1:1,000, Cell Signaling Technology, #4060), β-actin (1:5,000, Sigma-Aldrich, #5441).

### Animal Experiment (Tail Vein Injection)

Athymic male nu/nu mice aged from 5 weeks were used in this study. Lung metastasis model established by tail vein injection at a total cell number of 75 × 10^4^ for either shNC or shALDH1A3 HPAC cells in 300 μl PBS. Mice were sacrificed after five weeks. The entire lung tissue was collected. Then, lungs were fixed with 4% paraformaldehyde and analyzed with H&E staining. Area proportion of metastatic lesions on the largest section of each sample were calculated and analyzed. The animal experiments were approved by the Institutional Animal Care and Use Committee of the Affiliated Drum Tower Hospital of Nanjing University Medical School (20180102). All animal procedures were performed in compliance with the guidelines set by the Animal Care Committee, and all efforts were made to minimize potential pain and discomfort in the animals.

### Transwell Assay

The migration ability of PANC-1 cells transfected with control shRNA or ALDH1A3 was tested in a transwell Boyden chamber (8 mm). Cells were harvested and suspended in FBS-free DMEM culture medium at a concentration of 5 × 10^4^ cells/mL. DMEM medium (0.8 mL) containing 20% FBS was added to the lower compartment. 0.5 mL cell suspension was added to the upper chamber. Transwell-containing plates were incubated for 24 h in a 5% CO_2_ atmosphere saturated with H_2_O. Cells passing through the filter membrane were fixed with 4% paraformaldehyde at room temperature for 15 min, washed three times with distilled water, and stained with 0.5% crystal violet. We then gently removed the cells remaining on the upper surface of the filter membrane with a cotton swab. Images of the lower surfaces were captured using microscopy (five fields per chamber, CX31, Olympus) and the number of cells per field counted and analyzed. The experiments were performed three biological repeats and three technical replicates.

### RNA Sequence and GSEA

Total RNA was isolated and used for RNA-seq analysis. cDNA library construction and sequencing were performed by Beijing Genomics Institute using BGISEQ-500 platform. High-quality reads were aligned to the human reference genome (GRCh38). The expression levels for each of the genes were normalized to fragments per kilobase of exon model per million mapped reads (FPKM) using RNA-seq by Expectation-Maximization (RSEM).

Gene set enrichment analysis (GSEA) was performed on the Broad Institute Platform, and statistical significance (false discovery rate, FDR) was set at 0.25. Characteristic gene sets from control and ALDH1A3 overexpression groups were analyzed according to the genes presenting the strongest enrichment scores.

### Glucose and Lactate Measurement

A fluorescent glucose analog, 2-[N-(7-nitrobenz-2-oxa-1,3-diazol-4-yl) amino]-2-deoxy-glucose (2-NBDG, Sigma-Aldrich,#72987), was used to measure glucose uptake. Cells were seeded into 6-well-plate culture dishes for 24 h. Subsequently, 10 μM 2-NBDG was added to the culture medium, and the mixture was incubated for 20 min. To stop the response, cells were digested by trypsin followed by centrifuge and collected. The 2-NBDG fluorescence intensity was measured by flow cytometry at an excitation wavelength of 494 nm and an emission wavelength of 551 nm.

The culture medium was collected for measurement of lactate concentration. Lactate production in the medium was detected by using the Lactate Assay Kit (BioVision, #L256) according to the manufacturer's instruction. Total proteins were used for normalization of the results obtained above. The experiments were performed biologically and technically in triplicate and repeated twice.

### Extracellular ATP Measurement

The ATP levels were determined using a bioluminescent ATP assay kit (Beyotime, #S0027) according to the manufacturer's instruction. The standard ATP samples were used for the preparation of the calibration curve. Results were normalized by total protein from each sample. The experiments were performed biologically and technically in triplicate and repeated twice.

### Statistical Analyses

Data were analyzed using GraphPad Prism v7.0. All experiments were performed biologically and technically in triplicate, with the mean and standard deviation (SD) being reported where appropriate. And the repeated results were used as data points for statistical tests. ANOVA or Student's *t*-test was used for statistical analysis of differences in values among multiple groups. The differences in ALDH1A3 expression among patients were analyzed by *t*-test for continuous variables and the chi-squared test for categorical variables. Correlations were analyzed by the Pearson method. Log-rank tests were performed on Kaplan–Meier survival curves to determine any significant relationships between gene expression and patient outcomes. Differences were considered significant at *p* < 0.05.

## Data Availability Statement

The datasets generated for this study can be found in the NCBI BioProject [PRJNA608812].

## Ethics Statement

The studies involving human participants were reviewed and approved by the Ethical Committee of Medical Research, the Affiliated Drum Tower Hospital, Nanjing University, Medical School. The patients/participants provided their written informed consent to participate in this study. The animal study was reviewed and approved by the Institutional Animal Care and Use Committee of the Affiliated Drum Tower Hospital, Nanjing University, Medical School.

## Author Contributions

Study concept and design: SS, XZ, BK, SN. Acquisition of data: SN, CP, XD, SZ, BZ. Analysis and interpretation of data: SN, XQ, MS, HL. Drafting and editing of the manuscript: SN. Critical revision of the manuscript: GX, YL, LW, HF, BK. Administrative and material support, critical revision of the manuscript: SS, XZ. All authors gave final approval of the version to be published, and agree to be accountable for all aspects of the work.

## Conflict of Interest

The authors declare that the research was conducted in the absence of any commercial or financial relationships that could be construed as a potential conflict of interest.

## References

[1] Siegel RL Miller KD Jemal A Cancer statistics, 2020. CA Cancer J Clin. (2020) 70:7–30. 10.3322/caac.21590 31912902

[2] Makohon-Moore A Iacobuzio-Donahue CA. Pancreatic cancer biology and genetics from an evolutionary perspective. Nat Rev Cancer. (2016) 16:553–65. 10.1038/nrc.2016.66 27444064 PMC5739515

[3] Rahib L Smith BD Aizenberg R Rosenzweig AB Fleshman JM Matrisian LM. Projecting cancer incidence and deaths to 2030: the unexpected burden of thyroid, liver, and pancreas cancers in the United States. Cancer Res. (2014) 74:2913–21. 10.1158/0008-5472.CAN-14-0155 24840647

[4] Collisson EA Sadanandam A Olson P Gibb WJ Truitt M Gu S. Subtypes of pancreatic ductal adenocarcinoma and their differing responses to therapy. Nat Med. (2011) 17:500–3. 10.1038/nm.2344 21460848 PMC3755490

[5] Cheng T Jian Z Li K Raulefs S Regel I Shen S. *In vivo* functional dissection of a context-dependent role for Hif1α in pancreatic tumorigenesis. Oncogenesis. (2016) 5:e278. 10.1038/oncsis.2016.78 27941931 PMC5177776

[6] Kong B Bruns P Behler NA Chang L Schlitter AM Cao J. Dynamic landscape of pancreatic carcinogenesis reveals early molecular networks of malignancy. Gut. (2018) 67:146–56. 10.1136/gutjnl-2015-310913 27646934

[7] Kong B Cheng T Wu W Regel I Raulefs S Friess H. Hypoxia-induced endoplasmic reticulum stress characterizes a necrotic phenotype of pancreatic cancer. Oncotarget. (2015) 6:32154–60. 10.18632/oncotarget.5168 26452217 PMC4741665

[8] Kong B Wu W Cheng T Schlitter AM Qian C Bruns P. A subset of metastatic pancreatic ductal adenocarcinomas depends quantitatively on oncogenic Kras/Mek/Erk-induced hyperactive mTOR signalling. Gut. (2016) 65:647–57. 10.1136/gutjnl-2014-307616 25601637

[9] Kong B Cheng T Qian C Wu W Steiger K Cao J. Pancreas-specific activation of mTOR and loss of p53 induce tumors reminiscent of acinar cell carcinoma. Mol Cancer. (2015) 14:212. 10.1186/s12943-015-0483-1 26683340 PMC4683950

[10] Warburg O. On the origin of cancer cells. Science. (1956) 123:309–14. 10.1126/science.123.3191.309 13298683

[11] An MX Li S Yao HB Li C Wang JM Sun J. BAG3 directly stabilizes Hexokinase 2 mRNA and promotes aerobic glycolysis in pancreatic cancer cells. J Cell Biol. (2017) 216:4091–105. 10.1083/jcb.201701064 29114069 PMC5716268

[12] Jain M Nilsson R Sharma S Madhusudhan N Kitami T Souza AL. Metabolite profiling identifies a key role for glycine in rapid cancer cell proliferation. Science. (2012) 336:1040–4. 10.1126/science.1218595 22628656 PMC3526189

[13] Sreekumar A Poisson LM Rajendiran TM Khan AP Cao Q Yu J. Metabolomic profiles delineate potential role for sarcosine in prostate cancer progression. Nature. (2009) 457:910–4. 10.1038/nature07762 19212411 PMC2724746

[14] Mao P Joshi K Li J Kim SH Li P Santana-Santos L. Mesenchymal glioma stem cells are maintained by activated glycolytic metabolism involving aldehyde dehydrogenase 1A3. Proc Natl Acad Sci U S A. (2013) 110:8644–9. 10.1073/pnas.1221478110 23650391 PMC3666732

[15] Duan JJ Cai J Guo YF Bian XW Yu SC. ALDH1A3, a metabolic target for cancer diagnosis and therapy. Int J Cancer. (2016) 139:965–75. 10.1002/ijc.30091 26991532

[16] Panasyuk G Espeillac C Chauvin C Pradelli LA Horie Y Suzuki A. PPARγ contributes to PKM2 and HK2 expression in fatty liver. Nature Commun. (2012) 3:672. 10.1038/ncomms1667 22334075 PMC3293420

[17] Black W Vasiliou V. The aldehyde dehydrogenase gene superfamily resource center. Hum Genomics. (2009) 4:136–42. 10.1186/1479-7364-4-2-136 20038501 PMC3525204

[18] Ni W Xia Y Luo L Wen F Hu D Bi Y. High expression of ALDH1A3 might independently influence poor progression-free and overall survival in patients with glioma via maintaining glucose uptake and lactate production. Cell Biol Int. (2020) 44:569–82. 10.1002/cbin.11257 31642564

[19] Samson JM Ravindran Menon D Smith DE Baird E Kitano T Gao D. Clinical implications of ALDH1A1 and ALDH1A3 mRNA expression in melanoma subtypes. Chem Biol Interact. (2019) 314:108822. 10.1016/j.cbi.2019.108822 31580832 PMC6916670

[20] Li G Li Y Liu X Wang Z Zhang C Wu F. ALDH1A3 induces mesenchymal differentiation and serves as a predictor for survival in glioblastoma. Cell Death Dis. (2018) 9:1190. 10.1038/s41419-018-1232-3 30538217 PMC6290011

[21] Motomura H Nozaki Y Onaga C Ozaki A Tamori S Shiina TA. High Expression of c-Met, PKClambda and ALDH1A3 predicts a poor prognosis in late-stage breast cancer. Anticancer Res. (2020) 40:35–52. 10.21873/anticanres.13924 31892551

[22] Liu Y Wu K Shi L Xiang F Tao K Wang G. Prognostic significance of the metabolic marker hexokinase-2 in various solid tumors: a meta-analysis. PLoS One. (2016) 11:e0166230. 10.1371/journal.pone.0166230 27824926 PMC5100994

[23] Anderson M Marayati R Moffitt R Yeh JJ. Hexokinase 2 promotes tumor growth and metastasis by regulating lactate production in pancreatic cancer. Oncotarget. (2016) 8:56081–94. 10.18632/oncotarget.9760 28915575 PMC5593546

[24] Wullschleger S Loewith R Hall MN. TOR signaling in growth and metabolism. Cell. (2006) 124:471–84. 10.1016/j.cell.2006.01.016 16469695

[25] Edinger AL Thompson CB. Akt maintains cell size and survival by increasing mTOR-dependent nutrient uptake. Mol Biol Cell. (2002) 13:2276–88. 10.1091/mbc.01-12-0584 12134068 PMC117312

[26] Nakajima A Tomimoto A Fujita K Sugiyama M Takahashi H Ikeda I. Inhibition of peroxisome proliferator-activated receptor gamma activity suppresses pancreatic cancer cell motility. Cancer Sci. (2008) 99:1892–900. 10.1111/j.1349-7006.2008.00904.x 19016747 PMC11160097

[27] Hua TNM Namkung J Phan ANH Vo VTA Kim MK Jeong Y. PPARgamma-mediated ALDH1A3 suppression exerts anti-proliferative effects in lung cancer by inducing lipid peroxidation. J Recept Signal Transduct Res. (2018) 38:191–7. 10.1080/10799893.2018.1468781 29873276

[28] Nie S Huang Y Shi M Qian X Li H Peng C. Protective role of ABCG2 against oxidative stress in colorectal cancer and its potential underlying mechanism. Oncol Rep. (2018) 40:2137–46. 10.3892/or.2018.6594 30066914

[29] Shi M Duan G Nie S Shen S Zou X. Elevated FAM3C promotes cell epithelial- mesenchymal transition and cell migration in gastric cancer. Onco Targets Ther. (2018) 11:8491–505. 10.2147/OTT.S178455 30584315 PMC6287415

