# Supplementary material for: ALDH1A3 Accelerates Pancreatic Cancer Metastasis by Promoting Glucose Metabolism
Source: Front Oncol. 2020 Jun 16;10:915. doi: 10.3389/fonc.2020.00915 (PMC7308463; doi:10.3389/fonc.2020.00915)
Supplement: Supplementary file 1 [file Data_Sheet_1.DOCX]

**Supplementary method**

**Total ROS measurement**

DCFH-DA (Beyotime, S0033M) was used to measure total reactive oxygen species (ROS) produced in cells. Cells were seeded into 6-well plate culture dishes for 24 hours. Subsequently, 10 μM DCFH-DA was added to the culture medium, and the mixture was incubated for 20 min under 37℃. To stop the response, cells were digested by trypsin followed by centrifuge and collected. The fluorescence intensity was measured by flow cytometry at an excitation wavelength of 488 nm and an emission wavelength of 525 nm.

**Mito-ROS measurement**

Mito-SOX^TM^ (Sigma-Aldhrich, M36008) was used to measure reactive oxygen species (ROS) produced by mitochondria. Cells were seeded into 6-well plate culture dishes for 24 hours. Subsequently, 2.5 μM Mito-SOX^TM^ diluted with HBSS was added to cells, and the mixture incubated for 10 min under 37℃. To stop the response, cells were digested by trypsin followed by centrifuge and collected. The fluorescence intensity was measured by flow cytometry at an excitation wavelength of 510 nm and an emission wavelength of 580 nm.

**Supplementary Figure 1**. Overexpression of ALDH1A3 in PANC-1 cells decreased the production of ROS both in cells and in mitochondria.

1. The production of total ROS decreased in M24 and M31 cells compared to negative control cells.
2. ROS produced by mitochondria decreased in M24 and M31 cells compared to negative control cells.
